# Disruption of *gul*-*1* decreased the culture viscosity and improved protein secretion in the filamentous fungus *Neurospora crassa*

**DOI:** 10.1186/s12934-018-0944-5

**Published:** 2018-06-16

**Authors:** Liangcai Lin, Zhiyong Sun, Jingen Li, Yong Chen, Qian Liu, Wenliang Sun, Chaoguang Tian

**Affiliations:** Key Laboratory of Systems Microbial Biotechnology, Tianjin Institute of Industrial Biotechnology, Chinese Academy of Sciences, Tianjin, 300308 China

**Keywords:** *Neurospora crassa*, Mycelial morphology, Pellet, Viscosity, Protein secretion

## Abstract

**Background:**

The cellulolytic fungus *Neurospora crassa* is considered a potential host for enzyme and bioethanol production. However, large scale applications are hindered by its filamentous growth. Although previous investigations have shown that mycelial morphology in submerged culture can be controlled by altering physical factors, there is little knowledge available about the potential for morphology control by genetic modification.

**Results:**

In this study, we screened morphological mutants in the filamentous fungus *N. crassa*. Of the 90 morphological mutants screened, 14 mutants exhibited considerably higher viscosity compared with that of the wild type strain, and only two mutants showed low-viscosity morphologies in submerged culture. We observed that disruption of *gul*-*1* (NCU01197), which encodes an mRNA binding protein involved in cell wall remodeling, caused pellet formation as the fermentation progressed, and resulted in the most significant decrease in viscosity of culture broth. Moreover, over-expression of *gul*-*1* caused dramatically increased viscosity, suggesting that the *gul*-*1* had an important function in mycelial morphology during submerged cultivation. Transcriptional profiling showed that expression of genes encoding eight GPI-anchored cell wall proteins was lowered in Δ*gul*-*1* while expression of genes associated with two non-anchored cell wall proteins was elevated. Meanwhile, the expression levels of two hydrophobin genes were also significantly altered. These results suggested that GUL-1 affected the transcription of cell wall-related genes, thereby influencing cell wall structure and mycelial morphology. Additionally, the deletion of *gul*-*1* caused increased protein secretion, probably due to a defect in cell wall integrity, suggesting this as an alternative strategy of strain improvement for enzyme production. To confirm practical applications, deletion of *gul*-*1* in the hyper-cellulase producing strain (∆*ncw*-*1*∆*Ncap3m*) significantly reduced the viscosity of culture broth.

**Conclusions:**

Using the model filamentous fungus *N. crassa*, genes that affect mycelial morphology in submerged culture were explored through systematic screening of morphological mutants. Disrupting several candidate genes altered viscosities in submerged culture. This work provides an example for controlling fungal morphology in submerged fermentation by genetic engineering, and will be beneficial for industrial fungal strain improvement.

**Electronic supplementary material:**

The online version of this article (10.1186/s12934-018-0944-5) contains supplementary material, which is available to authorized users.

## Background

Filamentous fungi are widely used for industrial scale production of antibiotics, proteins and many other useful chemicals [1–3]. Unlike bacteria and yeast, filamentous fungi are morphologically complex microorganisms, which often leads to lots of process engineering problems during the fermentation process. When grown in submerged culture, fungi display different morphological forms, such as freely dispersed mycelia, clumps and pellets [4]. In general, the clump form predominates in fungal fermentations, resulting in high viscosity and a fermentation broth that shows several non-Newtonian characteristics. Although these disadvantages may not significant impact preliminary Erlenmeyer flask batch fermentations, when scaled up there are negative impacts on nutrient consumption and oxygen uptake, and a decrease in productivity. The simplest strategy to overcome this problem is to increase agitation speed. However, high impeller speed increases power consumption, and produces high shear stress that often damages fungal mycelia reducing product yield [5]. In contrast, the pellet growth form leads to Newtonian rheological behavior and low viscosities. As expected, lower power inputs are required for achieving sufficient agitation and mass transfer. Thus, the pellet morphology is preferred for many industrial processes, and it would greatly facilitate cost-effective production if one could design a way of controlling mycelial morphology during the fermentation process.

Many studies have reported on the effects of cultivation conditions and physical factors on fungal morphology, including media constituents, inoculum size, pH, temperature, agitation systems and the type of fermenter [5–8]. Since the genetics of morphogenesis is very complicated in filamentous fungi, there is little knowledge available about morphology control through genetic modification. In recent years, chemical and UV mutagenesis strategies have been widely used to create mutants that show low-viscosity morphology in submerged culture [9]. However, random mutagenesis is quite time-consuming, and it is also difficult to determine which gene is responsible for a desired phenotype.

In the past century, the filamentous fungus *Neurospora crassa* has been used as a model organism for genetic and molecular studies [10], leading to a range of genetic techniques and tools [11]. Compared with other filamentous fungi, only *N. crassa* possesses a near full genome deletion strain collection. Recently, *N. crassa* was deployed as a model to unravel mechanisms of lignocellulase expression and regulation due to its capacity to secrete lots of enzymes involved in lignocellulose utilization [12]. In addition, *N. crassa* has been reported to be a potential alternative candidate for heterologous protein expression and bioethanol production [13]. Several approaches have been employed to improve cellulase production, including regulation of the cellulase induction pathway, regulation of the unfolded protein response pathway, and by enhancing protein secretion [14–16]. Furthermore, *N. crassa* can be easily sexually crossed to generate multiple-gene mutants, which facilitates construction of hyper-cellulase producers. However, to our knowledge, *N. crassa* has not been used in industrial scale processes. One of the most limiting factors is its mycelial morphology in submerged culture. The *N. crassa* wild type strain exhibits a clump type of morphology, which results in high viscosity and mass transfer limitations. Although it is reported that *N. crassa* can grow as dispersed pellets following the addition of Triton N-101 or anionic polymer carboxypolymethylene [17, 18], investigations into the molecular controls of morphology could have great significance in industrial application.

Therefore, we screened 90 *N. crassa* morphological mutants to identify putative genes affecting mycelial morphology during submerged cultivation. Several candidate genes affected the viscosity of media when disrupted in *N. crassa*. Intriguingly, we found that disruption of *gul*-*1* (NCU01197), the homolog of *Saccharomyces cerevisiae ssd1*, led to a significant change in viscosity. In *S. cerevisiae*, SSD1 plays critical roles in maintaining cell wall integrity [19], and has been found to specifically associate with the mRNAs of proteins that are involved in cell wall remodeling [20]. SSD1 is also negatively regulated by the NDR family kinase Cbk1 through phosphorylation [21]. Deletion of *ssd1* alters the composition and cell wall architecture of the yeast cell surface [22]. Similar to the functions described for SSD1, GUL-1 is an important component of the NDR kinase COT-1 pathway, which is involved in the regulation of polarized growth in *N. crassa* [23, 24]. The NDR family kinase COT-1, which is a homolog of the *S. cerevisiae* Cbk1, is required for normal hyphal elongation [25]. The temperature-sensitive *cot*-*1* mutant exhibits cessation of tip extension and hyperbranching after being shifted to restrictive temperature. Disruption of *gul*-*1* can partially suppress the growth defects of *cot*-*1*. Thus, *gul*-*1* has been initially identified as a dominant modifier of *cot*-*1* [26]. Further investigation demonstrated that the Ste20 kinase POD-6 is involve in polar tip extension and also acts in the NDR kinase COT-1 pathway. The growth defects in *pod*-*6* and *cot*-*1* can be suppressed by the common extragenic suppressor, *gul*-*1*, suggesting that *gul*-*1* may function as a downstream mediator in this pathway [23]. Recently, Herold and Yarden provided strong evidence to support this hypothesis. Their results illuminated that the NDR family kinase COT-1 affects hyphal growth by influencing the transcript levels of cell wall remodeling genes, which is mediated by *gul*-*1* [24]. Despite the previous studies described above, the mechanism of how *gul*-*1* affects mycelial morphology in submerged culture remains unclear. In this study, we demonstrated that *gul*-*1* alters cell wall structure by influencing the expression of cell wall protein genes, which leading to changes in mycelial morphology. In addition, we also illustrated that morphology engineering enables enhanced protein secretion. Our findings enhance our understanding of the genetics of morphogenesis in filamentous fungi and provided a novel strategy for morphological engineering of filamentous fungi by genetic modification.

## Results

### A low-viscosity mutant from a morphological mutant library

**Fig. 1:**
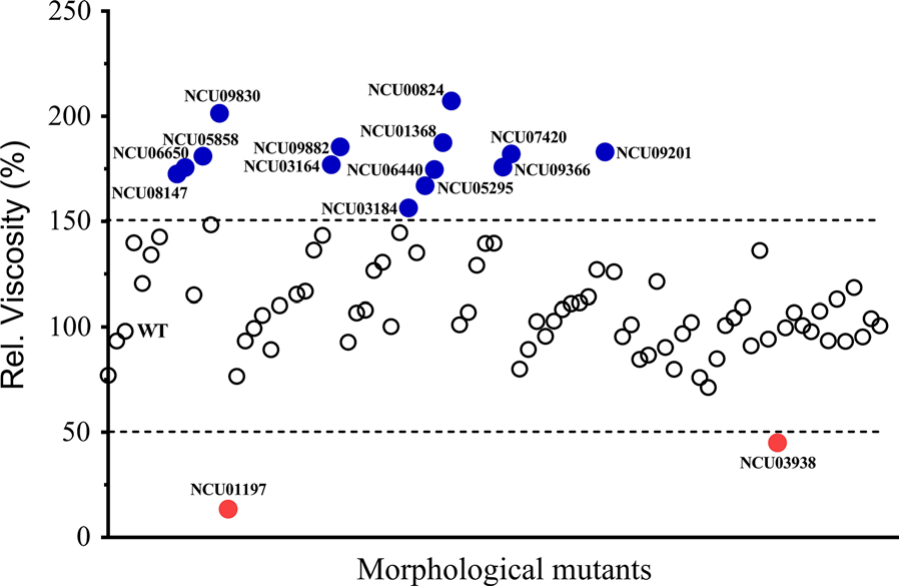
Screening of 90 morphological mutants in *Neurospora crassa*. Conidia from the wild type (WT) and morphological mutants were separately inoculated into Avicel medium and batch cultured for 7 days. The viscosities of culture broths altered by more than 50% compared with the WT are indicated as follows: blue dots, high-viscosity mutants; red dots, low-viscosity mutants

Colot et al. [27] constructed a nearly complete genome disruption mutant collection. There were two 96-well plates in this collection relevant to our study. One plate was annotated as “Morphologicals”, the other was identified as “Hyphal Growth Set” [28]. To identify the key genes that play important roles in mycelial morphology during submerged cultivation, we screened these 90 *N. crassa* morphological mutants. The viscosities of the fermentation broths are shown in Fig. 1 and Table 1. According to microscope observations, the majority of mutants resembled the wild type (WT) in growth characteristics. Compared to WT, 14 mutants showed dramatically increased viscosity (> 50%) and two mutants showed decreased viscosity (> 50%). Deletion of NCU03938 (encoding an alternative oxidase-5) reduced viscosity by approximately 55%, but this mutant still grew as clumps. Conversely, loss of NCU01197, which was annotated as *gul*-*1* previously [23], resulted in the pellet growth form and reduced viscosity by more than 80% compared with WT. Thus, *gul*-*1* was selected for further investigation.

**Table 1:** List of morphological mutants in *Neurospora crassa*

No.	NCU no.	Annotation	Viscosity (cP)	Increased vs WT (%)
1	NCU07075	Calcium exchanger	267.5 ± 3.5	− 22.9
2	NCU09866	Thyroid hormone receptor interactor 12	324.5 ± 34.6	− 6.5
3	NCU09423	Secreted protein	340.5 ± 12.0	− 1.9
4	NCU05854	Hypothetical protein	430.0 ± 127.3	23.9
5	NCU05790	Phytochrome-2	418.5 ± 9.2	20.6
6	NCU05956	Glycosylhydrolase family 2–2	466.0 ± 36.8	34.3
7	NCU07617	Aconidiate-3	407.5 ± 88.5	17.4
8	NCU08147	P-type ATPase	599.0 ± 58.0	72.6
9	NCU06650	Secretory phospholipase A2	610.0 ± 56.6	75.8
10	NCU06531	Hypothetical protein	458.0 ± 116.0	32.0
11	NCU05858	Fatty acid oxygenase	630.0 ± 42.4	81.6
12	NCU06419	MAPK/ERK kinase	495.0 ± 41.4	42.6
13	NCU09830	Menadione-induced gene-12	700.0 ± 28.3	101.7
14	NCU01197	Gulliver-1	42.0 ± 2.8	− 87.9
15	NCU09364	Heat shock protein 30	261.0 ± 26.9	− 24.8
16	NCU06265	Hypothetical protein	324.5 ± 54.4	− 6.5
17	NCU01213	Superoxide dismutase-2	345.0 ± 83.4	− 0.6
18	NCU09494	Hypothetical protein	366.5 ± 75.7	5.6
19	NCU08741	Hyphal anastomosis-3	380.0 ± 127.3	9.5
20	NCU03894	Serine/threonine protein kinase-4	382.5 ± 95.5	10.2
21	NCU01833	Nonidentical kinase-2	401.0 ± 79.2	15.6
22	NCU02542	Embden–meyerhof pathway-1	406.0 ± 19.8	17.0
23	NCU01181	Acyl-CoA dehydrogenase-3	473.5 ± 79.9	36.5
24	NCU08225	High affinity nickel transporter nic1	498.0 ± 46.7	43.5
25	NCU03164	Two-component system response regulator	615.0 ± 62.2	77.2
26	NCU09882	Metacaspase-1A	644.5 ± 21.9	85.7
27	NCU09450	Regulatory particle, non-ATPase-like-2	322.5 ± 64.3	− 7.1
28	NCU02260	Regulatory particle, ATPase-like-3	370.5 ± 43.1	6.8
29	NCU00634	Ribosomal protein L14	375.0 ± 77.8	8.1
30	NCU03702	rRNA 2′-*O*-methyltransferase fibrillarin	440.0 ± 67.9	26.8
31	NCU08050	Hypothetical protein	453.5 ± 50.2	30.7
32	NCU06764	Proteasome catalytic alpha-2	490.0 ± 155.6	41.2
33	NCU00396	Pre-mRNA-splicing factor rse-1	502.5 ± 51.6	44.8
34	NCU03184	C2H2 conidiation transcription factor FlbC	543.0 ± 28.3	56.5
35	NCU02604	Hypothetical protein	456.0 ± 33.9	31.4
36	NCU05295	Proteasome catalytic alpha-5	580.5 ± 26.2	67.3
37	NCU06440	Proteasome catalytic alpha-4	607.5 ± 41.7	75.1
38	NCU01368	Proteasome catalytic beta-4	651.5 ± 68.6	87.8
39	NCU00824	Histone deacetylase-3	720.0 ± 14.1	107.5
40	NCU06429	Alpha-actinin	351.0 ± 38.2	1.2
41	NCU00554	Homoserine-1	371.5 ± 64.3	7.1
42	NCU08093	Hypothetical protein	448.5 ± 78.5	29.3
43	NCU00105	60S ribosome subunit biogenesis protein NIP7	484.5 ± 17.7	39.6
44	NCU03479	Endoribonuclease ysh-1	485.0 ± 77.8	39.8
45	NCU09366	Proteasome catalytic beta-6	611.0 ± 55.2	76.1
46	NCU07420	eIF4A	633.0 ± 53.7	82.4
47	NCU00467	COP9 signalosome-5	267.5 ± 17.7	− 22.9
48	NCU01408	COP9 signalosome-3	310.5 ± 0.7	− 10.5
49	NCU00923	Topogenesis of outer membrane beta barrel protein 37	336.5 ± 19.1	− 3.0
50	NCU04669	Hypothetical protein	332.0 ± 72.1	− 4.3
51	NCU04242	Period-6	357.0 ± 42.4	2.9
52	NCU02057	Autoinducer 2 sensor kinase/phosphatase luxQ	376.0 ± 1.4	8.4
53	NCU09842	Mitogen activated protein kinase-1	385.5 ± 33.2	11.1
54	NCU01033	Hypothetical protein	387.0 ± 83.4	11.5
55	NCU08875	Cullin binding protein CanA	397.0 ± 49.5	14.4
56	NCU00810	Glycosylhydrolase family 2–3	436.5 ± 19.1	25.8
57	NCU09201	Hypothetical protein	636.0 ± 58.0	83.3
58	NCU04096	Protein kinase-9	462.9 ± 18.3	33.4
59	NCU00204	Hypothetical protein	306.3 ± 33.6	− 11.7
60	NCU00355	Catalase-3	331.1 ± 26.7	− 4.6
61	NCU01225	Ubiquitin conjugating enzyme	259.0 ± 29.8	− 25.4
62	NCU01312	Regulator of conidiation in Aspergillus-1	336.1 ± 33.8	− 3.1
63	NCU01613	Protoperithecia-2	372.0 ± 31.1	7.2
64	NCU01642	Hypothetical protein	283.8 ± 22.9	− 18.2
65	NCU02133	Superoxide dismutase-1	262.7 ± 24.5	− 24.3
66	NCU02387	Nuclear import and export protein Msn5	306.3 ± 33.6	− 11.7
67	NCU02498	Cullin-3	334.9 ± 21.4	− 3.5
68	NCU02794	Soft	269.0 ± 43.9	− 22.5
69	NCU03013	Anchored cell wall protein-10	277.7 ± 45.7	− 20.0
70	NCU03076	Delta-1-pyrroline-5-carboxylate dehydrogenase	305.0 ± 21.2	− 12.1
71	NCU03125	NIMA-interacting protein TinC	304.9 ± 21.0	− 12.1
72	NCU03281	Transport of copper-2	347.3 ± 18.0	0.1
73	NCU03314	mob2-like-a	364.6 ± 20.6	5.1
74	NCU03623	Ubiquitin-conjugating enzyme E	291.3 ± 12.4	− 16.1
75	NCU06175	Peroxin 3	473.0 ± 38.1	36.3
76	NCU06255	Hypothetical protein	317.4 ± 17.8	− 8.5
77	NCU03938	Alternative oxidase-5	156.9 ± 32.6	− 54.8
78	NCU04264	Extracellular developmental signal biosynthesis protein FluG	331.1 ± 26.7	− 4.6
79	NCU04302	Ubiquitin-conjugating enzyme E	331.0 ± 15.6	− 4.6
80	NCU04513	Ubiquitin conjugating enzyme Ubc14	364.6 ± 20.6	5.1
81	NCU04533	Abundant perithecial protein	339.9 ± 28.5	− 2.1
82	NCU04834	Phytochrome-1	373.4 ± 23.4	7.6
83	NCU05046	E1–E2 ATPase-1	329.7 ± 27.9	− 5.0
84	NCU05591	ABC transporter CDR4	353.3 ± 47.1	1.8
85	NCU07378	Serine/threonine protein kinase-12	323.7 ± 37.2	− 6.7
86	NCU07728	Siderophore regulation	416.9 ± 9.8	20.1
87	NCU08055	b-ZIP transcription factor IDI4	331.1 ± 26.7	− 4.6
88	NCU08791	Catalase-1	361.0 ± 26.9	4.0
89	NCU03725	Vegetative incompatibility blocked-1	415.7 ± 20.2	19.8
90	NCU03043	C2H2 finger domain-containing protein FlbC	356.0 ± 19.8	2.6

### GUL-1 is involved in mycelial morphology in *N. crassa*

In order to determine the extent of the morphological changes conferred by *gul*-*1* disruption, we conducted a microscopic analysis of Δ*gul*-*1* grown under permissive temperature. When grown on agar plates at 25 °C, there were significant differences in hyphal elongation and branching frequency between Δ*gul*-*1* and WT. The Δ*gul*-*1* exhibited slow growth rate and hyperbranching (Additional file 1: Figure S1), which is consistent with previous observations [23]. These results indicated that disruption of *gul*-*1* substantially impact hyphal development in *N. crassa*.

**Fig. 2:**
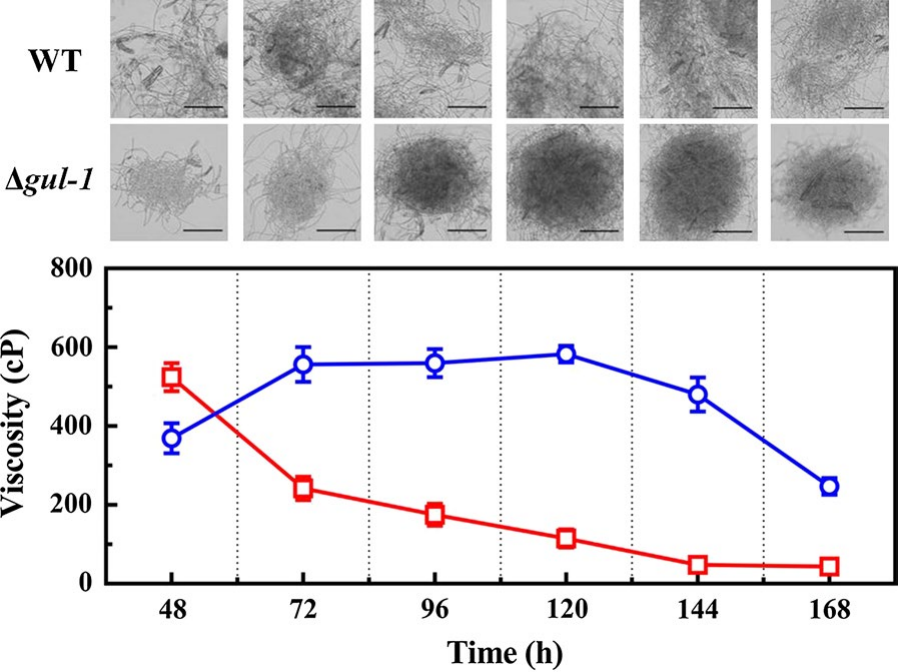
Mycelial morphologies of wild type and Δ*gul*-*1* mutant during submerged cultivation. Conidia from the wild type (WT) and *gul*-*1* mutant (Δ*gul*-*1*) were separately inoculated into Avicel medium and batch cultured for 7 days, and the viscosities of the broths were measured at 24 h intervals. The Δ*gul*-*1* mutant grew in pellet form, whereas the wild type exhibited a clump type morphology. Blue line indicates WT; Red line indicates the Δ*gul*-*1* mutant. Scale bar is 200 μm. Values represent the means of at least three replicates, error bars show standard deviation

A detailed analysis of the effects of disrupting *gul*-*1* on the rheological properties of mycelial broth was also performed. Conidia of WT and Δ*gul*-*1* were inoculated into Avicel medium and batch cultured for 7 days. The viscosity of WT increased to 557 ± 44 cp in the first 72 h before stabilizing until 120 h when the viscosity decreased significantly (Fig. 2). However, the rheological properties of Δ*gul*-*1* cultures were quite different from that of the WT. Firstly, the viscosity of Δ*gul*-*1* was much higher than that of WT 48 h post-inoculation, with its viscosity then decreasing dramatically during the time course of batch fermentation. After 7 days fermentation, the viscosity of Δ*gul*-*1* was only one-fifth that of WT (Fig. 2). According to microscopic observations, the WT showed a clump type of morphology. However, Δ*gul*-*1* grew as pellets that became more compact as fermentation progressed (Fig. 2). The diameter of pellets was about 350 ± 62 μm. Furthermore, scanning electron microscopy demonstrated that Δ*gul*-*1* exhibited much smoother surface than the WT (Additional file 2: Figure S2).

It is well established that mycelial biomass significantly affects the apparent viscosity of fermentation broth. Thus, the biomass of Δ*gul*-*1* and WT were determined as described [14]. The results revealed that the dry weight of Δ*gul*-*1* was similar to that of the WT (Additional file 3: Figure S3). Thus, the change in rheological properties was due to the morphological change in mycelia, and not to a reduction in biomass.

In addition, fungal morphology in submerged cultures is affected by the type of carbon source [29]. The Δ*gul*-*1* grew as pellets, and exhibited dramatically reduced viscosity (> 50%) compared with that of WT when grown on glucose, xylose and fructose (Additional file 4: Figure S4) indicated that growth form is independent of the type of carbon source. This suggests that *gul*-*1* might be considered as a universal target for morphology control under different cultivation conditions.

To the best of our knowledge, this is the first report indicating that *N. crassa* can change morphological forms in submerged culture (from clump type to pellet type) through genetic modification.

### Expression level of *gul*-*1* affected culture viscosity

**Fig. 3:**
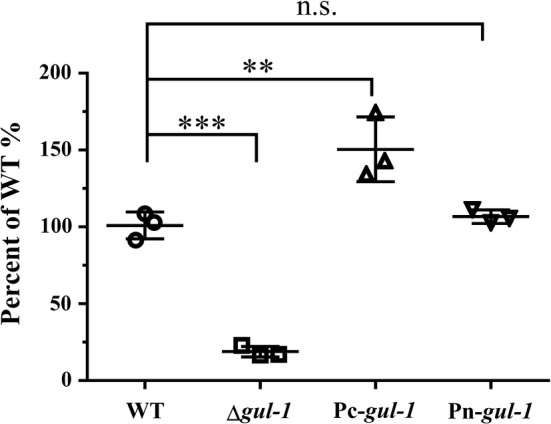
Comparison of the viscosity of culture broths from WT, Δ*gul*-*1*, Pc-*gul*-*1* and Pn-*gul*-*1* strains grown on Avicel medium for 7 days. The following strains were grown in 2% (w/v) Avicel media: the WT, the *gul*-*1* gene knockout mutant (Δ*gul*-*1*) and the complemented strain under either the control of the *ccg*-*1* promoter (Pc-*gul*-*1*) or the native promoter (Pn-*gul*-*1*). The viscosity was measured and displayed after normalization to the WT control according to percentage. Values represent the means of at least three biological replicates, error bars show standard deviation. Statistical significance was performed using a two-tailed Student’s *t* test (**P < 0.01; ***P < 0.001; n.s., not significant)

To determine whether *gul*-*1* expression levels affect the rheological properties of *N. crassa* fermentation, we engineered the Δ*gul*-*1* strain to express a C-terminal GFP-tagged GUL-1 under the regulation of either the native *gul*-*1* promoter (strain Pn-*gul*-*1*, complemented strain) or the constitutive *ccg*-*1* promoter (strain Pc-*gul*-*1*, *gul*-*1* over-expression strain). Complemented Pn-*gul*-*1* was indistinguishable from WT in any of the phenotypic assays performed in this study (data not shown). The Pc-*gul*-*1* strain showed WT growth rates on MM agar (Additional file 5: Table S1) and WT clump type morphology in submerged culture, but the viscosity was approximately 50% greater than that of WT (Fig. 3). These results indicate that *gul*-*1* plays important roles in controlling mycelial morphology during submerged cultivation.

### Subcellular localization of the GUL-1 protein in *N. crassa*

**Fig. 4:**
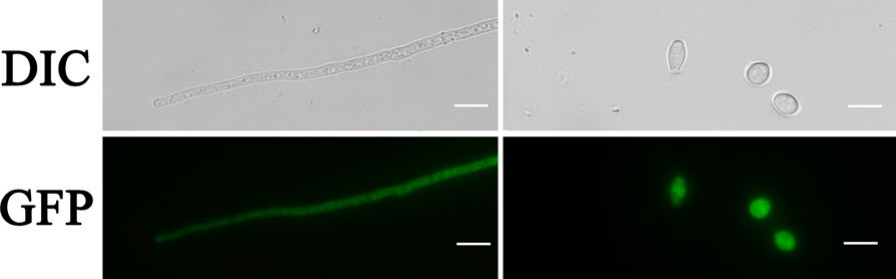
The subcellular localization of GUL-1 in *Neurospora crassa*. Locations of GUL-1 proteins were monitored by recording enhanced green fluorescent protein signal. Microscopic observation was performed with an OLYMPUS BX51 microscope. Scale bar is 10 μm

To assess the subcellular localization of GUL-1 protein, we tracked the C-terminal GFP-tagged GUL-1 in the Δ*gul*-*1* strain. Under its native promoter, GFP-tagged GUL-1 fluorescence was hard to observe in mycelia, but detectable in conidia (data not shown), indicating that *gul*-*1* had a lower expression level in mycelium than conidia. When the GUL-1-GFP fusion encoding gene was under the control of the *ccg*-*1* constitutive promoter, GFP fluorescence was uniformly distributed in the cytoplasm of both hyphae and conidia (Fig. 4), similar to that reported for the *Colletotrichum lagenarium* Classd1p [30]. However, recent studies have shown that *S. cerevisiae* Ssd1, the homolog of *N. crassa* GUL-1, transiently localizes to the nucleus [31]. To address whether GUL-1 also enters the nucleus, we surveyed GUL-1 for a signal sequence using NLStradamus which predicted a putative lysine-rich nuclear localization signal (amino acids 497–507: KREKEEKKKRK) [32]. Thus, GUL-1 is probable a nucleocytoplasmic protein, which usually localizes to the cytoplasm. The detailed mechanism of nucleocytoplasmic shuttling of GUL-1 remains to be elucidated.

### Sensitivity to cell wall inhibitors and oxidative agents

**Fig. 5:**
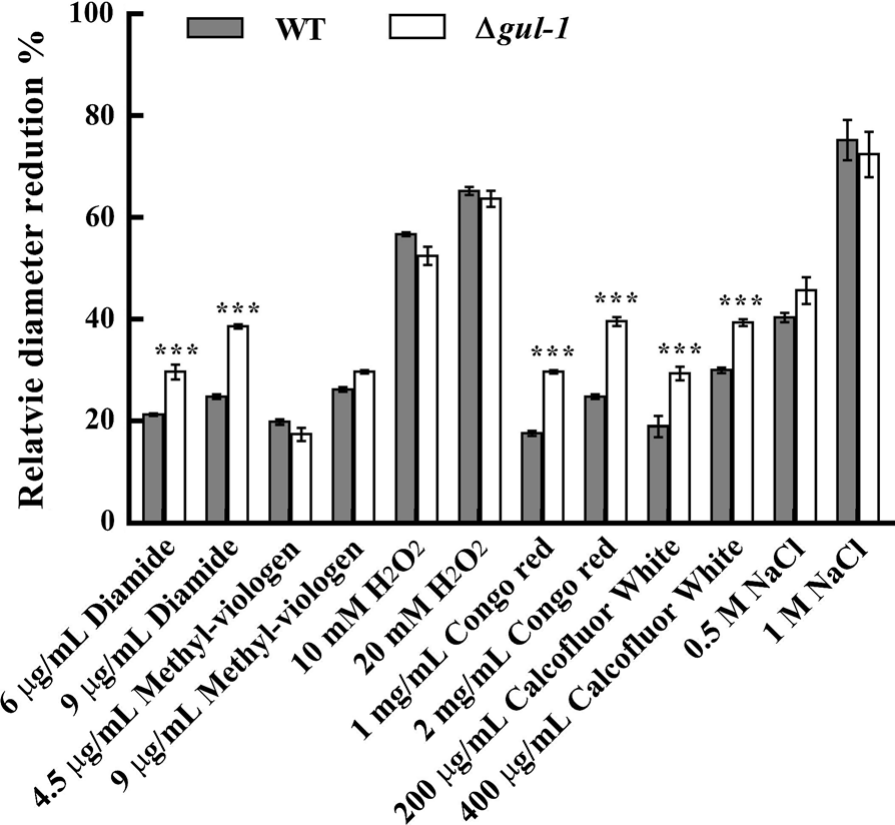
Effect of different concentrations of chemicals on hyphal growth in WT and Δ*gul*-*1*. Aliquots of 5 μL 1 × 10^7^ mL^−1^ spore suspensions of WT and Δ*gul*-*1* were incubated at 28 °C for 18 h on MM plates incorporating H_2_O_2_ (10, 20 mM), diamide (6, 9 μg/mL), methyl-viologen (4.5, 9 μg/mL), NaCl (0.5, 1.0 M), Congo Red (1, 2 mg/mL) or Calcofluor White (200, 400 μg/mL), and then the diameter of each colony was measured. Relative diameter reduction means the reduction of growth rate on MM containing chemicals compared with the growth on MM only. Values represent the means of at least three biological replicates, error bars show standard deviation. Statistical significance was performed using a two-tailed Student’s *t* test (***P < 0.001)

Our results indicate that *gul*-*1* influences the mycelial morphology of *N. crassa*, so we determined whether Δ*gul*-*1* suffers defects in cell wall integrity. We therefore examined its sensitivity to cell wall perturbation, oxidative stress and high osmolarity. The results showed that Δ*gul*-*1* had increased sensitivity to Congo Red, which binds to β-1,3-glucans and interferes with cell wall construction [33]. Growth of Δ*gul*-*1* on MM containing 1 mg/mL Congo Red was reduced by 29.7 ± 0.3%, whereas growth of WT was only reduced by 17.7 ± 0.5%. Likewise, Δ*gul*-*1* showed increased sensitivity to Calcofluor White, which disrupts cell wall synthesis by binding to chitin [34]. Growth of Δ*gul*-*1* on MM supplemented with 200 μg/mL Calcofluor White was reduced by over 29.3 ± 1.3%, whereas growth of WT was only reduced by 19.1 ± 2.1% (Fig. 5), suggesting that the mutant had altered cell wall integrity. These data are consistent with other studies, where deletion of the *gul*-*1* homolog in the plant pathogen *Magnaporthe grisea* also resulted in hypersensitivity to Calcofluor White [30].

Oxidative stress was provided by hydrogen peroxide (H_2_O_2_), diamide or methyl-viologen. These oxidants elicit oxidative damage to fungal cells in different ways. H_2_O_2_ is a common oxidative agent able to react with many biological molecules including proteins and DNA [35]. Diamide triggers oxidative stress by oxidizing intracellular glutathione [36]. Methyl-viologen is a redox cycling reagent that produces superoxide anions under aerobic condition [37]. All three oxidative agents partially inhibited the elongation of hyphae in both Δ*gul*-*1* and WT strains; only diamide was more effective on Δ*gul*-*1* than the WT (Fig. 5). The growth of Δ*gul*-*1* on MM agar containing 9 μg/mL diamide was reduced by 38.6 ± 0.4% as compared to 24.8 ± 0.5% for the WT. These results indicate that *gul*-*1* has a role in glutathione-mediated antioxidant processes, but not in general oxidative stress protection of *N. crassa*.

Δ*gul*-*1* grown on MM medium supplemented with NaCl exhibited no greater sensitivity to osmotic stress than the wild type strain (Fig. 5), indicating that *gul*-*1* is not required for osmotic stress tolerance.

### Deletion of *gul*-*1* improved protein secretion

**Fig. 6:**
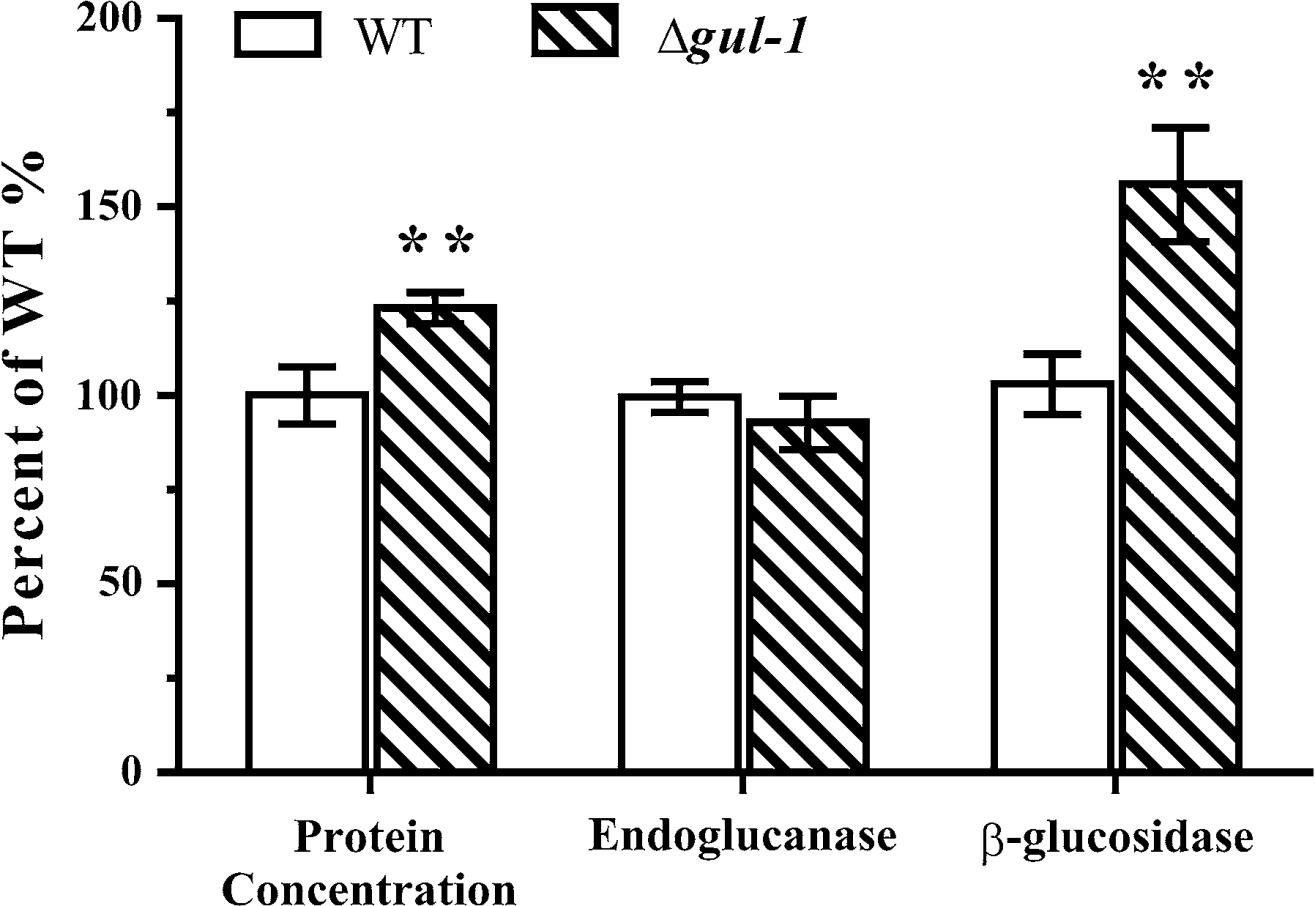
Phenotype of WT and Δ*gul*-*1* strains when grown on Avicel medium. Conidia from the wild type (WT) and the *gul*-*1* knockout mutant (Δ*gul*-*1*) were separately inoculated into Avicel medium and batch cultured. After 7 days, total extracellular protein concentration, endoglucanase activity and β-glucosidase activity were measured. Data were normalized to the WT control according to percentage. Values represent the means of at least three biological replicates, error bars show standard deviation. Statistical significance was performed using a two-tailed Student’s *t* test (**P < 0.01)

As a cellulolytic filamentous fungus, *N. crassa* possesses an extraordinary capacity to secret a wide variety of lignocellulolytic enzymes, making it a potential workhorse for production of industrial enzymes and high-value vaccines [38]. Based on our findings, we interrogated whether the defect in cell wall integrity would enhance protein secretion in *N. crassa*. The ability of WT and Δ*gul*-*1* to secrete cellulases was compared in Avicel medium. The Δ*gul*-*1* mutant secreted 25% more extracellular proteins 7 days post-inoculation. Although endoglucanase activity was unaffected, β-glucosidase activity in Δ*gul*-*1* was 56% higher than that of WT (Fig. 6). We also assessed whether disrupting *gul*-*1* would enhance protein secretion on other carbon sources. Compared with WT, the secreted protein titers in the Δ*gul*-*1* strain were 181, 57 and 43% higher than those of WT strain on sucrose, fructose or xylose, respectively (Additional file 4: Figure S4), suggesting that the enhancement of protein secretion in the Δ*gul*-*1* strain is independent of the carbon source. These results also indicate that cell wall integrity could play a critical role in protein secretion with implications for other filamentous fungi.

### Comparative analysis of transcriptomes of WT and the Δ*gul*-*1* strain

**Fig. 7:**
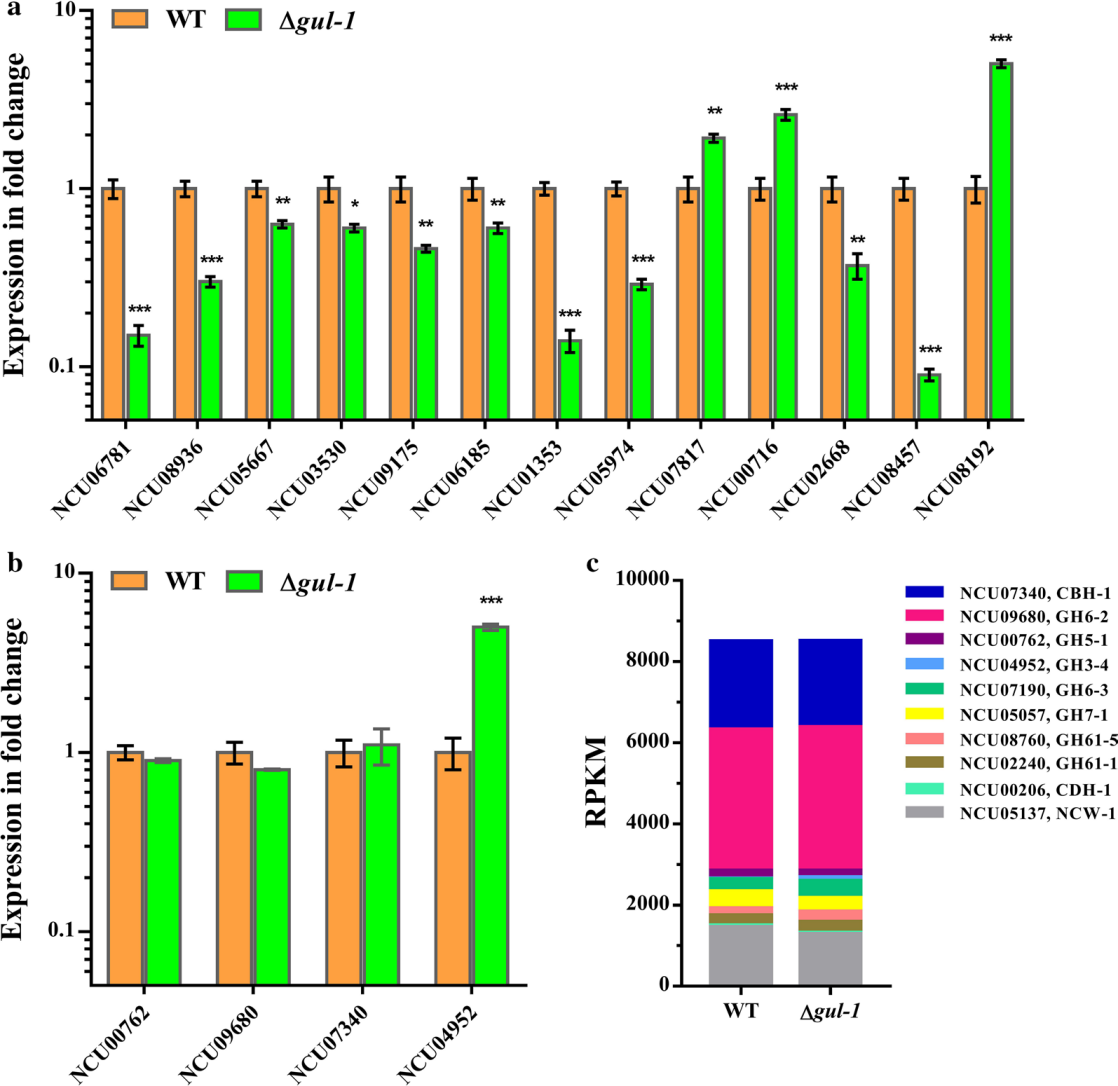
Transcriptome analysis of the Δ*gul*-*1* strain on Avicel medium. **a** Expression levels of genes encoding cell wall proteins in Δ*gul*-*1* mutant relative to wild-type (WT) strain on Avicel. **b** Expression levels of major cellulase genes in Δ*gul*-*1* mutant relative to the wild-type (WT) strain on Avicel. **c** Expression levels from RNA-seq data of genes encoding major secreted proteins from WT and Δ*gul*-*1* when grown on Avicel medium. After Δ*gul*-*1* and WT conidia were grown on Avicel for 3 days, the transcriptional abundance of major cellulase genes and cell wall protein genes was evaluated by RNA-seq and quantitative real-time PCR. Values represent the means of at least three biological replicates, error bars show standard deviation. Statistical significance was performed using a two-tailed Student’s *t* test (*P < 0.05, **P < 0.01, ***P < 0.001)

Disruption of *gul*-*1* in *N. crassa* caused pellet growth in submerged cultivation (Fig. 2). It is well established that fungal pellet formation is due to the combination of electrostatic interactions, hydrophobicity and specific interactions from cell wall components [39]. Based on the analysis above, we therefore hypothesized that the mechanism of pellet formation in Δ*gul*-*1* might feasibly be explained by changes in cell wall organization. To test this hypothesis, we evaluated transcriptional changes in the Δ*gul*-*1* mutant when grown on Avicel medium for 3 days using the WT as a control. RNA-seq data showed that the expression level of 101 genes was significantly lower in the Δ*gul*-*1* mutant, while the expression level of 145 genes increased (Additional file 6: Table S2). It is worth noting that the expression levels of several cell wall-related genes were significantly altered in Δ*gul*-*1*. These cell wall proteins are critical components of the fungal cell wall. Based on the manner in which they are attached to the cell wall polysaccharides, the cell wall proteins have been classified into two classes in *N. crassa*: GPI-anchored cell wall protein (ACW) and non-anchored cell wall protein (NCW) [40]. As previously reported, 22 cell wall proteins have been identified in *N. crass*a vegetative hyphae cell wall proteomic analysis [40]. Of the 22 identified genes, we detected 10 (45%) genes with altered expression (Fig. 7a and Additional file 7: Table S3). Eight GPI-anchored cell wall protein genes showed a decrease in expression level > twofold. Among these genes, four GPI-anchored glycoside hydrolase genes (NCU09175, NCU01353, NCU05974 and NCU6781), which were probably involved in cell wall remodeling, were down-regulated 2.1-, 7.1-, 3.4- and 6.7-fold in *gul*-*1* mutant, respectively, suggesting a reorganized arrangement of the cell wall. Moreover, anchored cell wall genes *acw*-*1* (NCU08936), *acw*-*3* (NCU05667), *acw*-*6* (NCU03530), and *acw*-*9* (NCU06185) were also significantly down-regulated in Δ*gul*-*1*. In *S. cerevisiae*, *acw*-*1* ortholog (*ecm33*) played a critical role in cell wall organization. Deletion of *ecm33* results in a weakened and disorganized cell wall [41]. On the contrary, non-anchored cell wall genes *ncw*-*3* (NCU07817) and *ncw*-*5* (NCU00716) were up-regulated 2.7- and 3.2-fold, respectively. Moreover, in *S. cerevisiae*, SUN4 is involved in cell wall remodeling, and regulated by the yeast GUL-1 homologue Ssd1 [21]. As expected, the cell wall synthesis gene (NCU02668), a homolog of *S. cerevisiae sun4*, was down-regulated 2.9-fold in Δ*gul*-*1*, suggesting a possible direct regulation of NCU02668 by GUL-1 in *N. crassa*. Furthermore, a previous study has demonstrated that hydrophobins play important roles in pellet formation. In *Aspergillus nidulans*, disruption of hydrophobin gene *rodA* led to a decrease in pellet biomass and size [42]. According to our results, the Class I hydrophobin gene *eas* (NCU08457) was down-regulated 15-fold in the Δ*gul*-*1* strain, whereas the Class II hydrophobin gene NCU08192 was up-regulated 6.2-fold, indicating that hyphal hydrophobicity might be changed in Δ*gul*-*1*. Taken together, the significant changes in expression of cell wall-related proteins suggested that the Δ*gul*-*1* mutant has an abnormal cell wall structure, which might be the reason for pellet formation.

In addition, the expression levels of major cellulase genes were not affected by the *gul*-*1* deletion, except that the extracellular β-glucosidase gene (NCU04952) had significantly higher expression level in Δ*gul*-*1* (Fig. 7b and c). These results are consistent with the enzyme activity assay shown in Fig. 6.

### Genetic engineering of *N. crassa* for hyper-cellulase production and lower viscosity

**Fig. 8:**
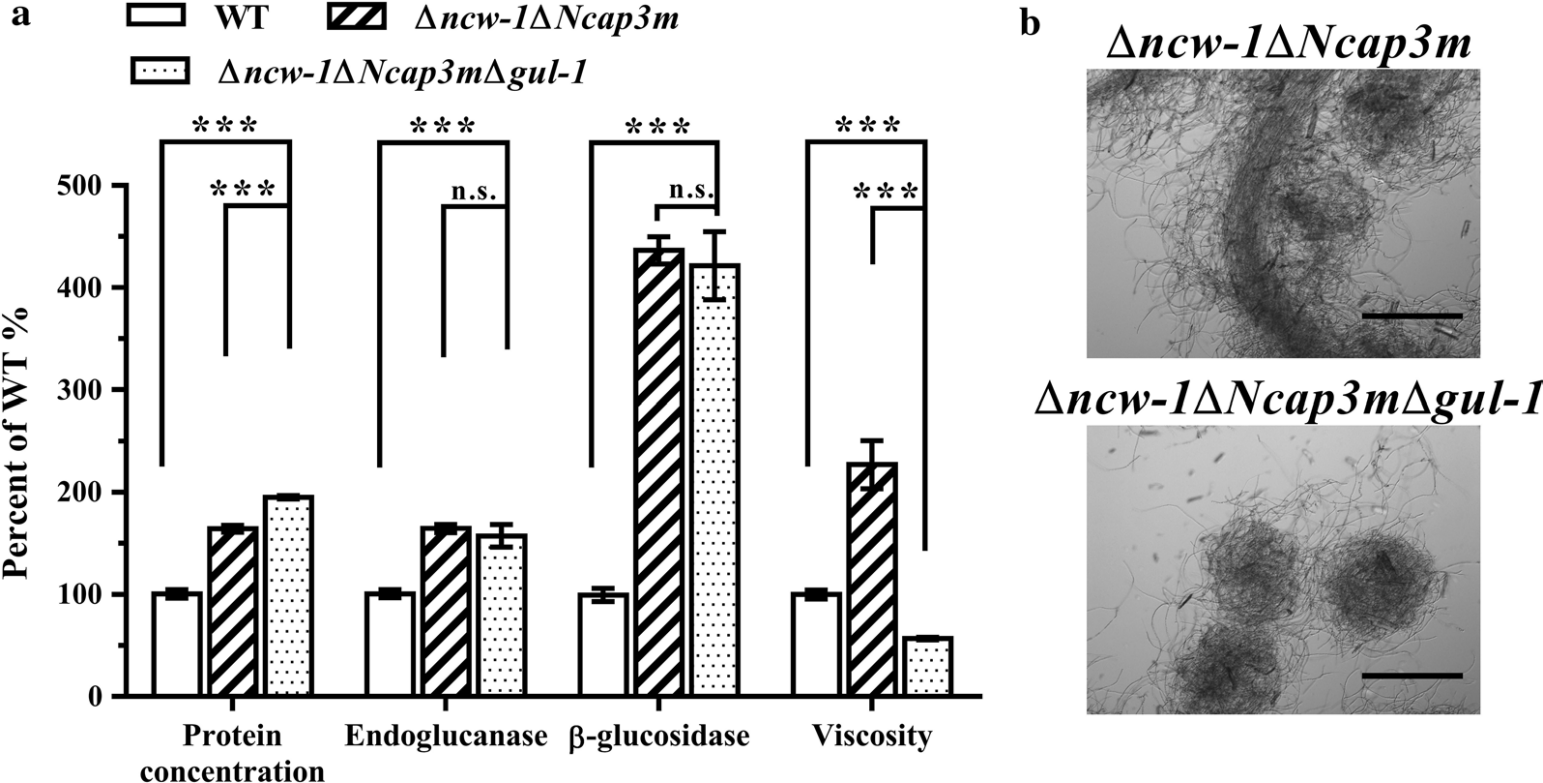
Phenotype of ∆*ncw*-*1*∆*Ncap3m*∆*gul*-*1* triple mutant when grown on Avicel medium. Conidia of the wild type (WT), the double deletion strain (∆*ncw*-*1*∆*Ncap3m*) and the triple deletion strain (∆*ncw*-*1*∆*Ncap3m*∆*gul*-*1*) were separately inoculated into Avicel medium, and then cultured for 7 days. **a** Total extracellular protein concentration, endoglucanase activity, β-glucosidase activity and the viscosity of culture broth were measured and normalized to the WT according to percentage. Values represent the means of at least three biological replicates, error bars show standard deviation. Statistical significance was performed using a two-tailed Student’s *t* test (***P < 0.001; n.s., not significant). **b** Mycelial morphologies of the triple deletion strain (∆*ncw*-*1*∆*Ncap3m*∆*gul*-*1*) and its parental strain (∆*ncw*-*1*∆*Ncap3m*) after 7 days cultivation. The images were acquired by an Olympus SZX-7 stereomicroscopy with a digital camera attached. Scale bar is 300 μm

Recent investigations have revealed that *N. crassa* is a good model system for unraveling the mechanisms of lignocellulose degradation [43, 44]. Meanwhile, several key genes involved in cellulase expression and secretion have been identified and characterized in *N. crassa*. NCU05137, which encodes a non-anchored cell wall protein (NCW-1), could be detected in the secreted proteome of cultures containing cellulose as sole carbon [45]. The disruption of *ncw*-*1* resulted in significantly increased cellulase production and cellulolytic activity [12]. In addition, we previously reported that disrupting *Ncap3m* (NCU03998, which encodes the μ subunit of the adaptor protein 3 complex) also dramatically increases lignocellulase secretion [16]. Based on these findings, a hyper cellulase producing strain ∆*ncw*-*1*∆*Ncap3m* was generated by sexual crosses. Compared to WT, the extracellular protein concentration of the ∆*ncw*-*1*∆*Ncap3m* strain was improved by approximately 65% (~ 0.8 g/L) and endoglucanase activity increased by about 73% (Fig. 8a). However, ∆*ncw*-*1*∆*Ncap3m* exhibited poor rheological properties, and the viscosity of ∆*ncw*-*1*∆*Ncap3m* was almost twofold higher than that of the WT. We therefore determined whether the rheological properties of the broth could be improved by deleting the *gul*-*1* gene in ∆*ncw*-*1*∆*Ncap3m*. The triple deletion strain (∆*ncw*-*1*∆*Ncap3m*∆*gul*-*1*) was obtained by crossing Δ*gul*-*1* with ∆*ncw*-*1*∆*Ncap3m*, and the progeny were verified by PCR. Not surprisingly, the triple deletion strain grew as pellets (Fig. 8b), and the viscosity of ∆*ncw*-*1*∆*Ncap3m*∆*gul*-*1* was only 23% that of the double deletion strain (∆*ncw*-*1*∆*Ncap3m*), and 53% that of the WT strain (Fig. 8a). Production of extracellular protein by ∆*ncw*-*1*∆*Ncap3m*∆*gul*-*1* was significantly improved relative to ∆*ncw*-*1*∆*Ncap3m*. However, no significant differences in endoglucanase and β-glucosidase activities were apparent.

## Discussion

In fungal fermentations, high apparent viscosity and non-Newtonian behavior of broths are critical problems in industrial application. Thus, controlling the morphology of filamentous fungus in submerged fermentations is a major challenge. Many strategies have been applied to solve these problems, principally by optimization of fermentation process parameters [5–8]. However, morphology control by altering chemical and physical factors is not sufficient. Moreover, these optimized parameters are not always suited for achieving maximum productivity. Recently, several attempts have been employed to control mycelial morphology by genetic modification. As previously reported, the motor proteins played critical roles in polarized growth in *Aspergillus* spp. Disruption of kinesin motor KipA in *Aspergillus glaucus* caused pellet growth in submerged cultivation and the aspergiolide A production was 82% higher than that of wild type strain [46]. Furthermore, disturbance of cell wall synthesis had significant influences on mycelial morphology [47]. In *Aspergillus orzyae*, disruption of *chsB* had no effect on enzyme production, but the viscosity was lower than that of parental strain [48]. In *Penicillium chrysogenum*, down-regulation of chitin synthase gene resulted in pellet growth in submerged culture, and also caused enhanced penicillin production [49]. Thus, rationally shaping mycelial morphology through genetic modification seems to be an attractive strategy. However, large-scale generation and screening of morphological mutants is difficult to achieve. Fortunately, *N. crassa* possesses a near full genome deletion strain collection [27]. In this work, we systematically screened the 90 *N. crassa* morphological mutants to identify the critical genes that could influence mycelial morphology during submerged cultivation. The viscosity of fermentation broth was used to reflect mycelial morphology change. Fourteen mutants showed increased viscosity compared to the WT strain. Among these mutants, we found several genes involved in the proteasome pathway, e.g., NCU01368, NCU05295, NCU09366 and NCU06440. All these four genes encode different subunits of the proteasome, implying that interruption of the protein degradation pathway might influence mycelial morphology. Moreover, NCU00824 encodes a histone deacetylase which possibly contributes to chromatin structure modification. Previous work in the yeast *C. albicans* has demonstrated that histone deacetylase interacting with transcription factors plays a critical role in morphological transition [50]. The high-viscosity phenotype induced by a defect in NCU00824 implies a close link between chromatin structure modification and morphology control in filamentous fungi.

Of most interest, we found that disruption of *gul*-*1* (NCU01197) in *N. crassa* led to favorable morphological changes in submerged culture. The Δ*gul*-*1* mutant grew as pellets and showed a low viscosity phenotype. Furthermore, scanning electron microscopy observations revealed that the cell wall structure was altered in the *gul*-*1* mutant. Our results with Congo Red and Calcofluor White also illustrated that *gul*-*1* is required for cell wall integrity. Based on these results, we hypothesized that pellet formation is closely associated with changes in cell wall structure. In filamentous fungi, β-(1,3)-glucan and chitin are two major structural components of the fungal cell wall. However, according to our results, the transcript levels of genes encoding chitin and glucan synthases (*chs1*-*7* and *gls*-*1*), which are involved in cell wall synthesis, were not changed by the *gul*-*1* deletion (Additional file 6: Table S2). Moreover, the expression levels of *chit*-*1* (NCU02184) and *gh18*-*5* (NCU04554), which are involved in cell wall maintenance and remodeling [51], were also not altered in Δ*gul*-*1* (Additional file 6: Table S2). It suggested that these genes are probably not involved in conferring the *gul*-*1* phenotype. On the other hand, cell wall proteins are the important components of the fungal cell wall, and also play important roles in cell wall biosynthesis and interactions with external environment [40]. Intriguingly, the transcript abundance of several cell wall protein genes was found to be significantly altered in Δ*gul*-*1*. Among these genes, the GPI-anchored cell wall β-1,3-endoglucanase gene *bgt*-*2* (NCU09175), which is seemingly involved in cell wall remodeling, was down-regulated 2.1-fold in Δ*gul*-*1*. A recent study also showed the cell wall architecture is altered in the Δ*bgt*-*2* mutant [52]. Likewise, the transcript abundances of GPI-anchored glycoside hydrolases NCU05974 and NCU06781, the homologs of *S. cerevisiae crh1* and *Aspergillus fumigatus gel2*, respectively, were also lower in Δ*gul*-*1*. It has been reported that both *S. cerevisiae crh1* and *A. fumigatus gel2* are required for normal cell wall structure. Loss of *S. cerevisiae crh1* causes hypersensitivity to Congo Red and Calcofluor White, suggesting defects in cell wall structure [53]. In *A. fumigatus*, the *gel2* disruption strain exhibits slower growth and an altered cell wall composition [54]. These implicated that down-regulation of both NCU05974 and NCU06781 may results in an abnormal cell wall structure, leading to influencing mycelial morphology during submerged cultivation. Besides that, other GPI-anchored cell wall proteins, which are of unknown function, are annotated as ACW (GPI-anchored cell wall protein) [40]. They may serve as important structural components of the wall, and play critical roles in maintaining cell wall stability and function [55]. For example, *AfuEcm33*, a homolog of the *N. crassa* GPI-anchored cell wall protein-1 (*acw*-*1*), has been shown to be involved in maintaining cell wall integrity and virulence in *A. fumigatus* [56]. It is worth noting that disruption of *AfuEcm33* results in increased cell–cell adhesion. The disruption strain forms large ‘star-shaped’ clusters of germinating conidia during submerged cultivation [56]. In *Candida albicans*, a similar aggregate phenotype has been also observed in the *Caecm33* deletion strain [57]. In this study, the GPI-anchored cell wall protein ACW-1 encoded by NCU08936 was found to be down-regulated in Δ*gul*-*1*, suggesting that ACW-1 might, at least in part, contribute to pellet formation by increased cell–cell adhesion. Taken together, our results indicated that *gul*-*1* affects mycelial morphology during submerged cultivation by influencing the expression of cell wall-related protein. However, a detailed mechanistic description of how the *gul*-*1* influences these genes expression requires further investigation.

According to our results, the deletion of *gul*-*1* led to a hypersecretion phenotype in *N. crassa*. When grown on 2% (w/v) Avicel, the extracellular protein secreted by the Δ*gul*-*1* mutant was about 25% higher than that in the WT, while only β-glucosidase activity was found to be improved. These results were also confirmed by our transcriptomic analysis (Fig. 7b and c). However, the increase in activity was disproportionate to the increase in total protein, suggesting that the effect of *gul*-*1* on protein secretion is not due to increased transcription levels. Thus, we hypothesized that the defects in cell wall integrity benefit protein secretion. According to the ‘bulk flow’ hypothesis, growing hyphal tips are filled with vesicles that contain extracellular proteins, and these proteins get carried to the outer region of the wall for secretion [58]. However, some extracellular proteins might become trapped in the rigid cell wall. These ‘trapped’ enzymes could be released into an external medium through a weakening of cell wall integrity [59], which may explain the Δ*gul*-*1* mutant having a higher protein secretion capacity. Similar results could be observed in the *N. crassa slime* mutant which has serious defects in cell wall synthesis [60].

Our previous study demonstrated that the disruption of *ncw*-*1* led to enhanced cellulase production in *N. crassa*, but unfortunately the viscosity of ∆*ncw*-*1* was increased two fold [14]. A similar phenotype was also observed in the ∆*ncw*-*1*∆*Ncap3m* strain. Having identified the impact of *gul*-*1* disruption on mycelial morphology during submerged cultivation, we applied this approach to the hyper-cellulase producing strain ∆*ncw*-*1*∆*Ncap3m*. The triple deletion strain (∆*ncw*-*1*∆*Ncap3m*∆*gul*-*1*) grew in pellet form and exhibited a low viscosity phenotype, indicating that *gul*-*1* is a promising target gene for morphological improvement of hyper-cellulase-producing strains. In addition, phylogenetic analyses demonstrated that GUL-1 is highly conserved in other industrial filamentous fungi, including *Aspergillus niger* (XP_001392470, identity 66%), *Myceliophthora thermophila* (XP_003661716, 75% identity) and *Trichoderma reesei* (XP_006964501, 78% identity) (Additional file 8: Figure S5). Thus, manipulation of *gul*-*1* homologs could be a highly useful strategy for morphology engineering of industrial filamentous fungi.

## Conclusions

In this work, we systematically screened morphological mutants of *N. crassa* and identified several potential genes that affect mycelial morphology in submerged cultivation. Deletion of one of these genes, *gul*-*1*, resulted in the pellet form and enhanced protein secretion in *N. crassa*. Transcriptional profiling revealed that a deletion of *gul*-*1* led to an alteration in transcription of genes encoding cell wall proteins, which may be the reason for changes in mycelial morphology during submerged cultivation. Furthermore, a significant reduction in viscosity of broth culture was obtained when *gul*-1 was deleted from the hyper cellulolytic strain ∆*ncw*-*1*∆*Ncap3m*. These results indicated that *gul*-*1* might be a potential target to control mycelial morphology in submerged cultivation. This work provides a novel strategy for morphological engineering of filamentous fungi through genetic modification, especially for industrially relevant species.

## Methods

### Strains, growth conditions and microscopy

*Neurospora crassa* strains were obtained from Fungal Genetics Stock Centre (FGSC), including wild type (WT, FGSC 2489, A), a *his*-*3* mutant strain (FGSC 9716, *his*-*3*, *a*) and the morphological mutants. The *his*-*3*; Δ*gul*-*1*, a strain was obtained by crossing FGSC 9716 with FGSC 11288 (NCU01197, A). Multiple deletion strains were made by performing sequential crosses. All of the constructed strains were verified by PCR.

To obtain conidia, *N. crassa* was grown on slant tubes containing Vogel’s minimal media (1 × Vogel’s salts, 2% w/v sucrose and 1.5% w/v agar) for 10 days at 28 °C. For flask culture, 10-day-old conidia of *N. crassa* strains were suspended in sterile water and inoculated using a final concentration of 1 × 10^5^ per mL into a 250-mL Erlenmeyer flask containing 100 mL Avicel medium (1 × Vogel’s salts, 2% w/v Avicel, 0.75% w/v yeast extract and 0.2% v/v Tween 80), then cultured for 7 days at 25 °C, with shaking at 200 rpm under constant light. The observations were carried out on 7 consecutive days at 24 h intervals.

Microscopic observations were carried out using an OLYMPUS BX51 microscope equipped with a QImagingRetiga 2000R camera (QImaging, Surrey, Canada), and analyzed with the Image-Pro Express 6.3 software. The images were also acquired by an Olympus SZX-7 stereomicroscopy with a digital camera attached.

To compare the differences in cell surface structure between the WT and Δ*gul*-*1*, fungal conidia were incubated in Avicel medium at 25 °C for 5 days. The harvested mycelia were fixed in 0.1 M phosphate buffer (pH 7.2) containing a final concentration of 2.5% (w/v) glutaraldehyde. The samples were rinsed in thrice-distilled PBS, and dehydrated with a series of ethanol solutions (30–100%, v/v). After which the samples were dried by critical point drying (Leica CPD300) and coated with platinum (Hitachi E-1045). Observations were conducted using a scanning electron microscope (Hitachi SU8010) operating at 1 kV.

### Plasmid construction and transformation

Genomic DNA from the WT strain was extracted as described previously [61]. To complement Δ*gul*-*1*, the open reading frame (ORF) of *gul*-*1* was cloned by PCR with primers ORF-F and ORF-R, and the fragment was inserted into the *Xba*I and *Pac*I sites of pMF272 to form pMF272-Pc-gul-1-gfp. The promoter of *gul*-*1* was amplified with primers PF and PR, and then cloned into the *Not*I and *Xba*I sites of pMF272-Pc-gul-1-gfp to form pMF272-Pn-gul-1-gfp. The primers used in this study are listed in Additional file 9: Table S4. All PCR products in this study were cloned into the pGEM-T vector (Promega) and sequenced for confirmation.

Plasmid DNA (10 μg) was transformed into a (*his*-3; Δ*gul*-*1*, a) strain as described previously [16]. The constructs were targeted to the *his*-*3* locus by homologous recombination, and correct integration was confirmed by GFP fluorescence and PCR. To recover homokaryotic strains, His^+^GFP^+^ transformants were crossed with FGSC 11288 (NCU01197, A). Progeny were selected for histidine prototrophy and GFP fluorescence. The resulting complemented strains were termed Pc-*gul*-*1* and Pn-*gul*-*1*.

### Enzyme activity assay

For protein assays, 5 mL of culture supernatants were collected at each time point, centrifuged at 12,000 rpm for 10 min to remove mycelia and stored at 4 °C for further analysis. Total extracellular proteins were quantified using a Bio-Rad Protein Assay kit based on absorbance at 595 nm, with bovine serum albumin as the standard. The endoglucanase activity was determined using the azo-CMC assay kit according to manufacturer’s instruction (Megazyme). We used *p*-nitrophenyl-β-d-glucopyranoside (*p*NPG) for the measurement of β-glucosidase activity as described previously [62]. Briefly, a 250 μL culture supernatant that was diluted with 50 mM sodium citrate (pH 4.8) was added to 250 μL of 1 mg/mL *p*NPG, and then incubated immediately for 10 min at 50 °C. The reaction mixture was terminated by adding 500 μL of 1 M Na_2_CO_3_ and determined at an absorbance of 420 nm. Inactive enzyme, which was boiled at 100 °C for 10 min, was used as a control. *p*NP was used to generate a standard curve. One unit (U) of β-glucosidase activity was defined as the amount of *p*NP released from the substrate per minute using 1 mL enzyme under the standard assay conditions.

### Fungal biomass assay and rheological measurement

The dry weight of fungal biomass was indirectly measured as previously described [14]. Briefly, a 5-mL culture broth was centrifuged at 4000 rpm for 10 min, the supernatant was discarded, and the pellet was dried and weighed. The dried residue was resuspended in 3 mL of acetic nitric reagent (80:20, v/v), and bathed with boiling water for 2 h to remove mycelia. The residual Avicel was washed thoroughly, then dried, and reweighed. Mycelial dry weight was defined as the dry weight of the original 5-mL culture minus that of residual Avicel.

Rheological properties of fermentation broth were measured with a Brookfield digital HBDV-III Ultra rheometer according to the manufacturer’s instructions. Viscosity was measured as the torque exerted by the mycelial sample on the rheometer impeller at 50 rpm. Torque was recorded after the reading had stabilized (45–60 s).

### Oxidative stress, high osmotic stress and cell wall integrity test

For plate assays, 5 μL aliquots of conidial suspensions (10^7^ mL^−1^) were applied onto Vogel’s solid medium (MM: 1 × Vogel’s salts, 2% w/v sucrose and 1.5% w/v agar) supplemented with H_2_O_2_ (10, 20 mM), diamide (6, 9 μg/mL), methyl-viologen (4.5, 9 μg/mL), NaCl (0.5, 1.0 M), Congo Red (1, 2 mg/mL) or Calcofluor White (200, 400 μg/mL). Plates were incubated at 28 °C for 18 h. All colonies were cross-measured to determine their growth indices under each stress.

### RNA extraction, sequencing and data analysis

For gene expression analysis, conidia of ∆*gul*-*1* and WT strains were separately inoculated into Avicel medium (1 × Vogel’s salt, 2% w/v crystalline cellulose, 0.75% w/v yeast extract and 0.2% v/v Tween 80) and batch cultured for 3 days at 25 °C with constant light. Mycelia were harvested by vacuum filtration and frozen immediately in liquid nitrogen. Total RNA from frozen samples was isolated using Trizol reagent (Invitrogen) in accordance with the manufacturer’s protocol, and further treated with DNase I (RNeasy Mini Kit, Qiagen). The RNA integrity was checked by agarose gel electrophoresis. RNA-seq was performed on the Illumina HiSeqTM 2000 platform of Beijing Genomics Institute (BGI, Shenzhen, China). Filtered clean reads were mapped against predicted transcripts from the *N. crassa* OR74R genome (version 12) [63] using TopHat (version 2.0.8b) [64]. The alignment results were stored in SAM format files for subsequent analysis. The counts of reads that uniquely mapped to only one gene were calculated for each gene by Htseq-count (http://www-huber.embl.de/users/anders/HTSeq) using SAM files and genome annotation as input. Abundance for each transcript was calculated using the reads per kilobase per million (RPKM) [65]. Genes with altered expression were performed by using R package NOISeq (version 2.6.0) [66] (Q value ≥ 0.95 used as threshold, which approximately corresponds to a |log_2_ ratio| ≥ 1). To discover significantly upregulated and downregulated genes between WT and Δ*gul*-*1*, only the genes with relative high transcriptional abundance (RPKM values above 20 in at least one strain) went into further analysis [67]. Expression differences detected by RNA-seq were also verified by RT-PCR. Profiling data are listed in Additional file 6: Table S2, and the sequence data produced in this study can be accessed (GEO: GSE113321).

### Quantitative real-time PCR

Quantitative real-time PCR (qRT-PCR) was performed using a CFX96 real-time PCR detection system (Bio-Rad) with reagents from TOYOBO (One-step qPCR Kit). All assays were performed in triplicate with actin (NCU04173) as the endogenous standard, according to the manufacturer’s instruction. All primers used in this study were listed in Additional file 9: Table S4.

### Phylogenetic analysis

Putative orthologs of *gul*-*1* in selected fungal species were identified as best reciprocal BLAST hits from the National Center for Biotechnology Institute (NCBI) protein database. The phylogenetic analysis was carried out using the Neighbor-Joining in MEGA4 software. Bootstrap values were adjacent to each internal node, representing the percentage of 1000 bootstrap replicates.

### Statistical analyses

Unless otherwise noted, all experiments were performed in triplicate and statistical tests for significance were determined by two-tailed Student’s *t* test.

## Additional files

**Additional file 1: Figure S1.** Mycelial morphology of Δ*gul-1* and wild type strains. Cultures were grown on Vogel’s minimal medium for 18 h at 28 °C. Scale bar is 500 μm.

**Additional file 2: Figure S2.** Scanning electron micrographs of hyphal morphology of wild type and Δ*gul-1* strains. Cultures were grown on Avicel medium for 5 days at 25 °C. Scale bar is 10 μm.

**Additional file 3: Figure S3.** Biomass accumulation of WT and Δ*gul-1* mutant when grown on Avicel medium. Conidia from Δ*gul-1* and wild type (WT) strains were separately inoculated into Avicel medium and batch cultured for 7 days. The biomass accumulation was measured. Values represent the means of at least three replicates, error bars show standard deviation.

**Additional file 4: Figure S4.** Phenotype of WT and Δ*gul-1* strains when grown on different carbon sources. (A) Viscosity; (B) Fungal morphology; (C) Protein secretion. Conidia were inoculated into 100 mL liquid media [1×Vogel’s salts, 0.75% w/v yeast extract, 0.2% v/v Tween 80 and 2% w/v carbon source (sucrose, xylose or fructose)] at 10^5^ conidia/mL and grown at 25 °C in constant light and shaking (200 rpm). Statistical significance was performed using a two-tailed Student’s *t*-test (*P<0.05) Scale bar is 1500 μm.

**Additional file 5: Table S1.** Growth rates of wild type and *gul-1* mutant. Aliquots of 5 μL 1×10^7^ mL^−1^ spore suspensions of WT, Δ*gul-1*, Pn-*gul-1* and Pc-*gul-1* were incubated at 28 °C for 18 h on MM plates.

**Additional file 6: Table S2.** Gene list of the 246 genes differentially expressed in Δ*gul-1* as compared to WT on Avicel.

**Additional file 7: Table S3.** Comparative analysis of cell wall-related gene expression between Δ*gul-1* and WT by RNA-seq.

**Additional file 8: Figure S5.** Phylogenetic analysis of GUL-1 and its homologs. MEGA 4 software was used to carry out the analysis. Bootstrap values are adjacent to each internal node, representing the percentage of 1,000 bootstrap replicates.

**Additional file 9: Table S4.** Primers used in this study.
