# Supplementary figures and images for: Disruption of gul-1 decreased the culture viscosity and improved protein secretion in the filamentous fungus Neurospora crassa
Source: Microb Cell Fact. 2018 Jun 16;17:96. doi: 10.1186/s12934-018-0944-5 (PMC6004096; doi:10.1186/s12934-018-0944-5)

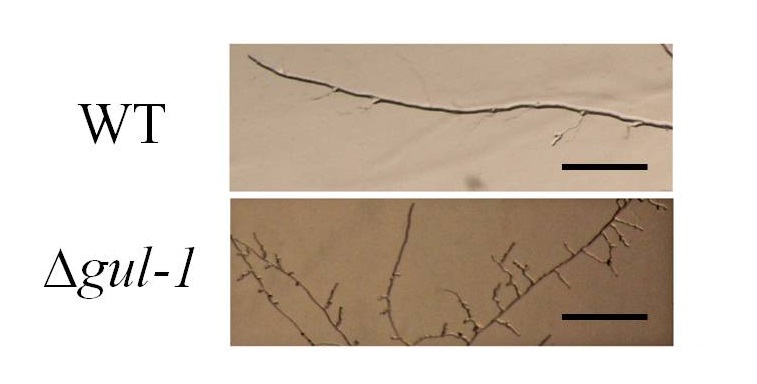

Supplement: Supplementary file 1 — Additional file 1: Figure S1. Mycelial morphology of Δgul-1 and wild type strains. Cultures were grown on Vogel’s minimal medium for 18 h at 28 °C. Scale bar is 500 μm. [file 12934_2018_944_MOESM1_ESM.jpg]

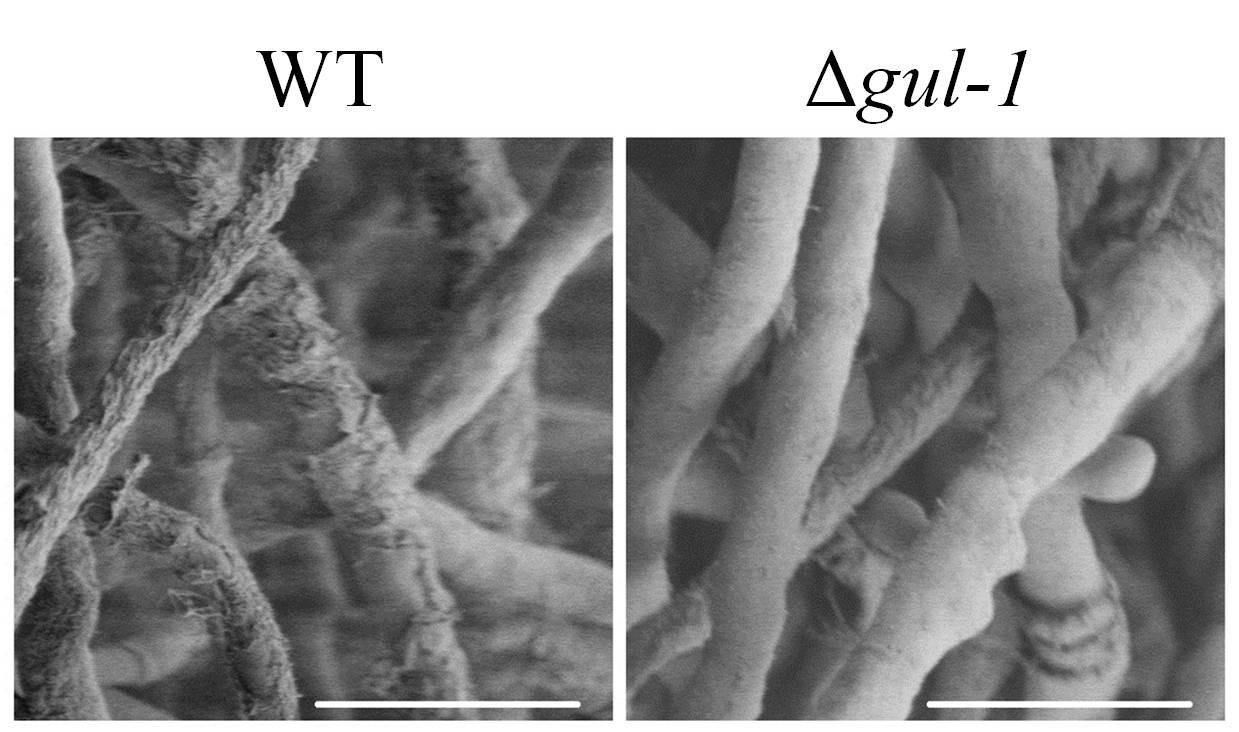

Supplement: Supplementary file 2 — Additional file 2: Figure S2. Scanning electron micrographs of hyphal morphology of wild type and Δgul-1 strains. Cultures were grown on Avicel medium for 5 days at 25 °C. Scale bar is 10 μm. [file 12934_2018_944_MOESM2_ESM.jpg]

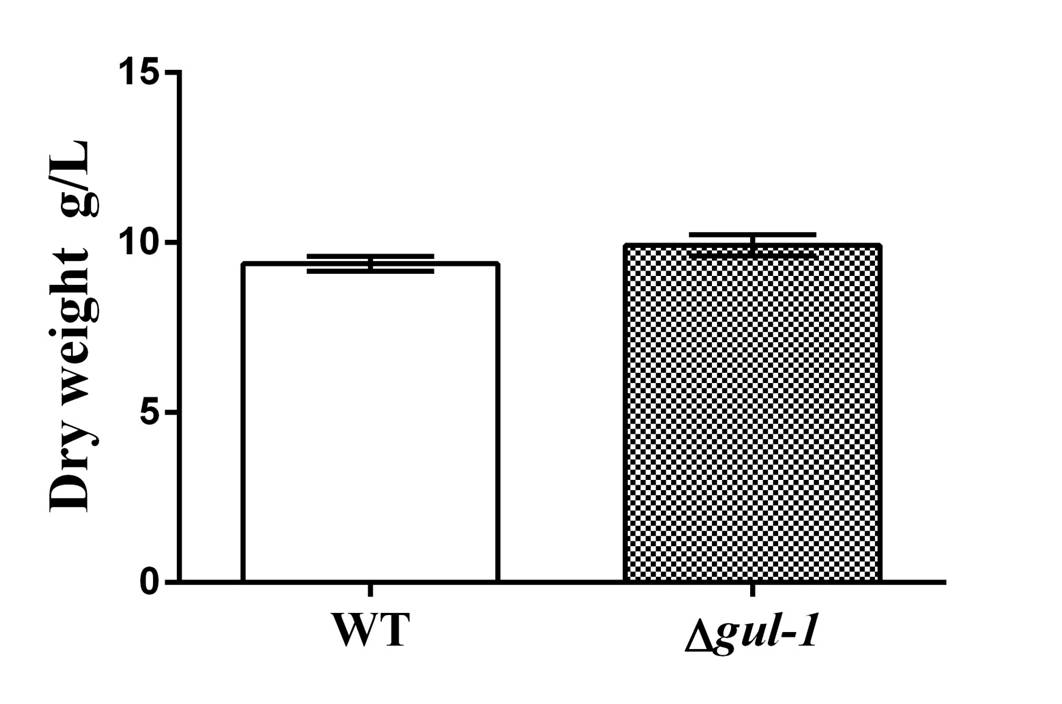

Supplement: Supplementary file 3 — Additional file 3: Figure S3. Biomass accumulation of WT and Δgul-1 mutant when grown on Avicel medium. Conidia from Δgul-1 and wild type (WT) strains were separately inoculated into Avicel medium and batch cultured for 7 days. The biomass accumulation was measured. Values represent the means of at least three replicates, error bars show standard deviation. [file 12934_2018_944_MOESM3_ESM.jpg]

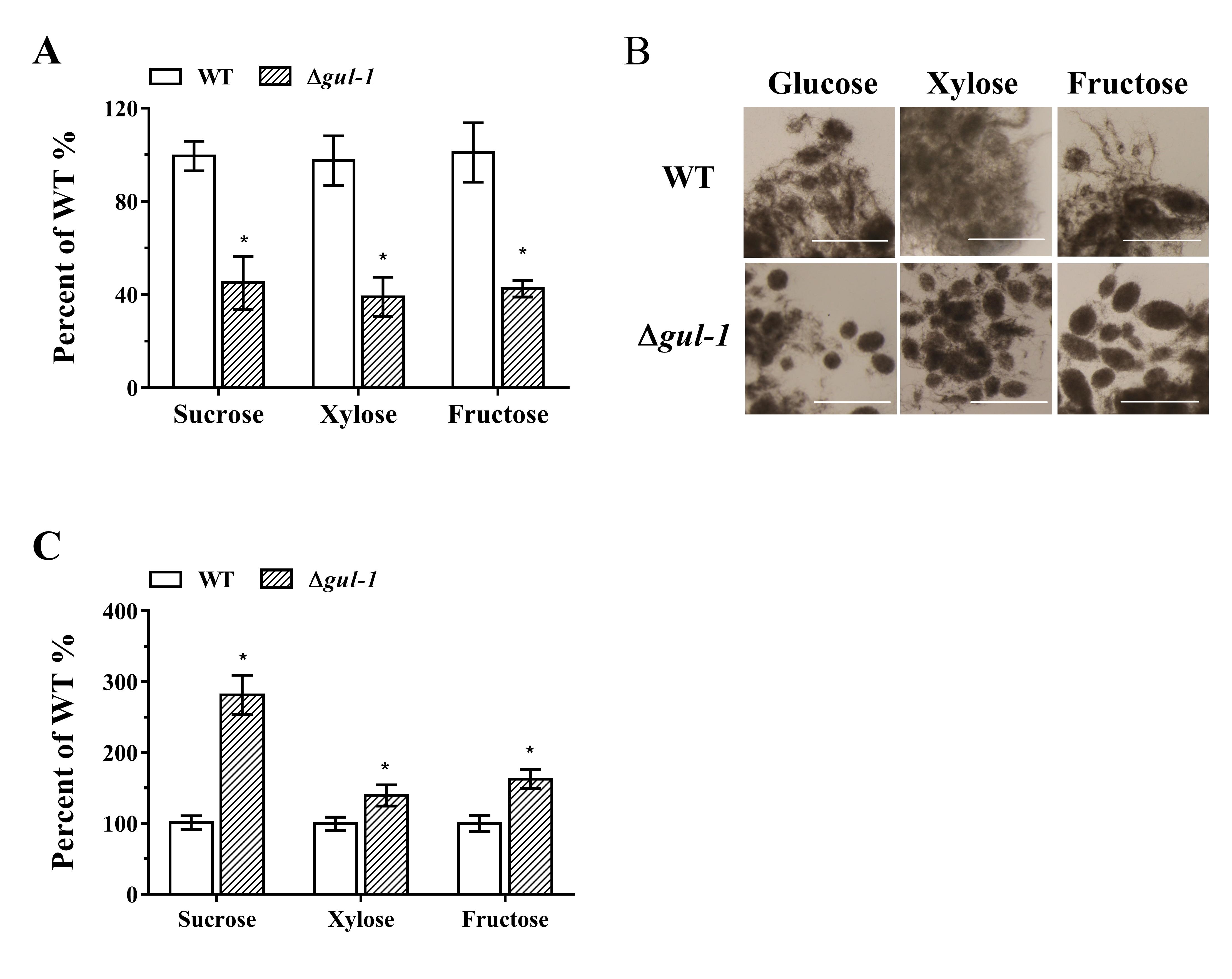

Supplement: Supplementary file 4 — Additional file 4: Figure S4. Phenotype of WT and Δgul-1 strains when grown on different carbon sources. (A) Viscosity; (B) Fungal morphology; (C) Protein secretion. Conidia were inoculated into 100 mL liquid media [1×Vogel’s salts, 0.75% w/v yeast extract, 0.2% v/v Tween 80 and 2% w/v carbon source (sucrose, xylose or fructose)] at 105 conidia/mL and grown at 25 °C in constant light and shaking (200 rpm). Statistical significance was performed using a two-tailed Student’s t-test (*P<0.05) Scale bar is 1500 μm. [file 12934_2018_944_MOESM4_ESM.jpg]

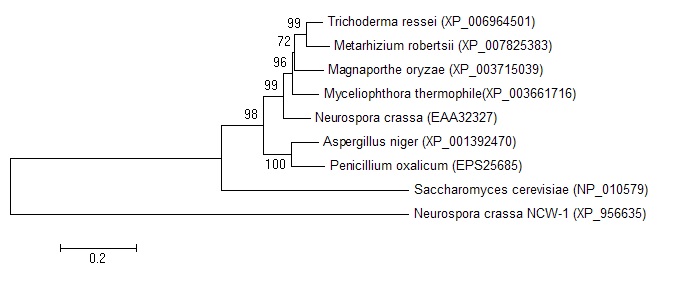

Supplement: Supplementary file 8 — Additional file 8: Figure S5. Phylogenetic analysis of GUL-1 and its homologs. MEGA 4 software was used to carry out the analysis. Bootstrap values are adjacent to each internal node, representing the percentage of 1,000 bootstrap replicates. [file 12934_2018_944_MOESM8_ESM.jpg]
