# Supplementary material for: Disruption of gul-1 decreased the culture viscosity and improved protein secretion in the filamentous fungus Neurospora crassa
Source: Microb Cell Fact. 2018 Jun 16;17:96. doi: 10.1186/s12934-018-0944-5 (PMC6004096; doi:10.1186/s12934-018-0944-5)
Supplement: Supplementary file 5 — Additional file 5: Table S1. Growth rates of wild type and gul-1 mutant. Aliquots of 5 μL 1×107 mL−1 spore suspensions of WT, Δgul-1, Pn-gul-1 and Pc-gul-1 were incubated at 28 °C for 18 h on MM plates. [file 12934_2018_944_MOESM5_ESM.docx]

**Table S1** Growth rates of wild type and *gul-1* mutant

| Strain | Wild type | ∆*gul-1* | Pn-*gul-1* | Pc-*gul-1* |
| --- | --- | --- | --- | --- |
| Growth rate (cm/d) | 5.81±0.86 | 3.70±0.63 | 5.97±0.81 | 5.93±0.92 |
