# Supplementary material for: Disruption of gul-1 decreased the culture viscosity and improved protein secretion in the filamentous fungus Neurospora crassa
Source: Microb Cell Fact. 2018 Jun 16;17:96. doi: 10.1186/s12934-018-0944-5 (PMC6004096; doi:10.1186/s12934-018-0944-5)
Supplement: Supplementary file 7 — Additional file 7: Table S3. Comparative analysis of cell wall-related gene expression between Δgul-1 and WT by RNA-seq. [file 12934_2018_944_MOESM7_ESM.docx]

Table S3 Comparative analysis of cell wall-related gene expression between Δ*gul-1* and WT by RNA-seq

| Protein name ^a^ | Gene Locus No. ^b^ | Fold change (Δ*gul*-1/ WT) ^c^ |
| --- | --- | --- |
| ACW-1 | NCU08936 | **2.3↓** |
| ACW-2 | NCU00957 | nc |
| ACW-3 | NCU05667 | **2.2↓** |
| ACW-5 | NCU07776 | nc |
| ACW-6 | NCU03530 | **2.2↓** |
| ACW-7 | NCU09133 | nc |
| ACW-8 | NCU07277 | nc |
| ACW-9 | NCU06185 | **2.3↓** |
| ACW-10 | NCU03013 | nc |
| ACW-11 | NCU02041 | nc |
| GH17-3 | NCU09175 | **2.5↓** |
| GH16-3 | NCU01353 | **5.8↓** |
| GH16-7 | NCU05974 | **2.5↓** |
| CHIT-1 | NCU02184 | nc |
| β-glucosidase | NCU09326 | nc |
| GH55-3 | NCU07253 | nc |
| GH72-2 | NCU06781 | **2.3↓** |
| NCW-1 | NCU05137 | nc |
| NCW-2 | NCU01752 | nc |
| NCW-3 | NCU07817 | **2.7↑** |
| NCW-5 | NCU00716 | **3.1↑** |
| CAT-1 | NCU00355 | nc |
| Hydrophobin | NCU08192 | **6.3↑** |
| EAS | NCU08457 | **15.7↓** |

^a^ abbreviations: ACW (GPI-anchored cell wall protein), NCW (non-anchored cell wall protein), GH (glycoside hydrolase), CAT (catalase), EAS (easily wettable).

^b^ Based on version 12 annotation of the Broad Institute's *Neurospora crassa* database.

^c^ up-regulated genes, bold red; down-regulated genes, bold green; nc, not change.
