# Supplementary material for: Disruption of gul-1 decreased the culture viscosity and improved protein secretion in the filamentous fungus Neurospora crassa
Source: Microb Cell Fact. 2018 Jun 16;17:96. doi: 10.1186/s12934-018-0944-5 (PMC6004096; doi:10.1186/s12934-018-0944-5)
Supplement: Supplementary file 9 — Additional file 9: Table S4. Primers used in this study. [file 12934_2018_944_MOESM9_ESM.docx]

Table S4 Primers used in this study

| Name | Sequence (5’-3’) |
| --- | --- |
| NCU01197DCUP | CCTCTTTCCGACCACCGT |
| NCU01197DCDown | AAGAGGGGGATGAGGGGT |
| NCU05137DCUP | GAGACACACACACGACTTGGAT |
| NCU05137DCDown | CCGAAGTTGGTCTGGTAGGT |
| NCU03998DCUp | AGCAGTCTTCGCTAAGGAACATC |
| NCU03998DCDown | GGTGAAAGGAAAAAGAGAGAGAT |
| NCUDCUP | GCTCCGGGCGTATATGCT |
| ORF-F: | TTCTTCTAGAATGGATCAGCAACAGCAGCCTC |
| ORF-R | TTCTTTAATTAAGAGGGCATAGGGATTGAGCGAG |
| PF | TTCTGCGGCCGCGCATCAGTTCGTCACTAGCAT |
| PR | TTCTTCTAGAGATGCAGGTTGTTGGGACGGA |
| 7340qF | GATGCCTACCAGGGCTTCG |
| 7340qR | GTGTACTTGGCCTTGCCACC |
| 9680qF | CTCTGCCTGGAGCCAGTGC |
| 9680qR | GGAAGAAGCCCCAGCAGTG |
| 762qF | GAGTTCACATTCCCTGACA |
| 762qR | CGAAGCCAACACGGAAGA |
| 4952qF | AACAAGGTCAACGGTACGTGG |
| 4952qR | TCGTCATATCCATACCACTGTTTG |
| 1353qF | TCACCTCCGTCATTGCCATCTC |
| 1353qR | TCACCATTGCCATTGCCATTGC |
| 5974qF | AGTACCAGTATGGCGACAAGAGC |
| 5974qR | AGGTGGAGGAGGCGGAAGG |
| 8457qF | GCTGCGTTGTCGGTGTCATC |
| 8457qR | GCGTTGATGATGAGGAAGGAGTTG |
| 9175qF | GTACTACTGGCAGGAGGTTGGC |
| 9175qR | GGTGGAGAGGTTGTCGGTGATAG |
| 6185qF | TTCTCCGCTGCTGCTCTTGTC |
| 6185qR | CGAGGTGACGACGGTGCTG |
| 5667qF | ACGACAAGAAGGAGACCACCAAG |
| 5667qR | GAGGCAGCAGCGGAGGAAG |
| 3530qF | GTCACCTCCGACGCTTCCTC |
| 3530qR | CGACACCAGAGATGCCAGAGATG |
| 8192qF | TGGAGCGTCAAGTCCCTTACAC |
| 8192qR | TCGAAGTGGGTGGCGTTGG |
| 716qF | ACCGTCAACCAGTGCGAGTTC |
| 716qR | CCGTGGCATCTGACCGAGTC |
| 2668qF | CCAGTCCACCACCGCTCAG |
| 2668qR | CCATCGTCACTCTTGCCAACAC |
| 6781qF | ACTGTCAAGAACGCCGATGGTAG |
| 6781qR | GCCCGAGGTGGTGGAGTTG |
| 8936qF | CACCATCTCCGACAGCCATCTC |
| 8936qR | TCGCCAGTGATCTCCTTGATAGC |
| 7817qF | GTGGTGGCTTCGTCAATCTGAAC |
| 7817qR | CAGGAGTGCAGGCGGTCAAG |
| actin_qF | TGATCTTACCGACTACCT |
| actin_qR | CAGAGCTTCTCCTTGATG |

The underlined regions represent the restriction sites.
